# 4-(4-Iodo­anilino)-2-methyl­ene-4-oxo­butanoic acid

**DOI:** 10.1107/S160053681205012X

**Published:** 2012-12-15

**Authors:** Prakash S. Nayak, Badiadka Narayana, Hemmige S. Yathirajan, Thomas Gerber, Benjamin van Brecht, Richard Betz

**Affiliations:** a Mangalore University, Department of Studies in Chemistry, Mangalagangotri 574 199, India; b University of Mysore, Department of Studies in Chemistry, Manasagangotri, Mysore 570 006, India; c Nelson Mandela Metropolitan University, Summerstrand Campus, Department of Chemistry, University Way, Summerstrand, PO Box 77000, Port Elizabeth, 6031, South Africa

## Abstract

In the title compound, C_11_H_10_INO_3_, an addition product of itaconic acid anhydride and 4-iodo­aniline, the least-squares planes defined by the atoms of the aromatic moiety and the non-H atoms of the carb­oxy­lic acid group enclose an angle of 74.82 (11)°. In the crystal, classical O—H⋯O hydrogen bonds formed by carb­oxy­lic groups, as well as N—H⋯O hydrogen bonds formed by amide groups, are present along with C—H⋯O contacts. Together, these connect the mol­ecules into dimeric chains along the *b-*axis direction.

## Related literature

For applications of itaconic acid anhydride, see: Oishi (1980 ▶); Urzua *et al.* (1998 ▶); Shetgiri & Nayak (2005 ▶); Katla *et al.* (2011 ▶); Hanoon (2011 ▶). For graph-set analysis of hydrogen bonds, see: Etter *et al.* (1990 ▶); Bernstein *et al.* (1995 ▶).
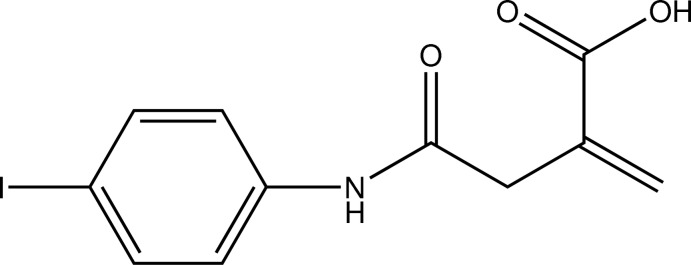


## Experimental

### 

#### Crystal data

C_11_H_10_INO_3_
*M* *_r_* = 331.10
Monoclinic, 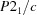

*a* = 23.1111 (6) Å
*b* = 4.7863 (1) Å
*c* = 10.4262 (2) Å
β = 97.071 (1)°
*V* = 1144.54 (4) Å^3^
*Z* = 4
Mo *K*α radiation
μ = 2.79 mm^−1^
*T* = 200 K
0.29 × 0.16 × 0.10 mm

#### Data collection

Bruker APEXII CCD diffractometer
Absorption correction: multi-scan (*SADABS*; Bruker, 2008 ▶) *T*_min_ = 0.503, *T*_max_ = 0.776
12435 measured reflections
2839 independent reflections
2612 reflections with *I* > 2σ(*I*)
*R*_int_ = 0.015

#### Refinement

*R*[*F* ^2^ > 2σ(*F* ^2^)] = 0.018
*wR*(*F* ^2^) = 0.047
*S* = 1.06
2839 reflections
150 parameters
H atoms treated by a mixture of independent and constrained refinement
Δρ_max_ = 0.73 e Å^−3^
Δρ_min_ = −0.61 e Å^−3^

### 

Data collection: *APEX2* (Bruker, 2010 ▶); cell refinement: *SAINT* (Bruker, 2010 ▶); data reduction: *SAINT*; program(s) used to solve structure: *SHELXS97* (Sheldrick, 2008 ▶); program(s) used to refine structure: *SHELXL97* (Sheldrick, 2008 ▶); molecular graphics: *ORTEP-3* (Farrugia, 2012 ▶) and *Mercury* (Macrae *et al.*, 2008 ▶); software used to prepare material for publication: *SHELXL97* (Sheldrick, 2008 ▶) and *PLATON* (Spek, 2009 ▶).

## Supplementary Material

Crystal structure: contains datablock(s) I, global. DOI: 10.1107/S160053681205012X/gk2533sup1.cif

Supplementary material file. DOI: 10.1107/S160053681205012X/gk2533Isup2.cdx

Structure factors: contains datablock(s) I. DOI: 10.1107/S160053681205012X/gk2533Isup3.hkl

Supplementary material file. DOI: 10.1107/S160053681205012X/gk2533Isup4.cml

Additional supplementary materials:  crystallographic information; 3D view; checkCIF report

## Figures and Tables

**Table 1:** Hydrogen-bond geometry (Å, °)

*D*—H⋯*A*	*D*—H	H⋯*A*	*D*⋯*A*	*D*—H⋯*A*
O1—H1⋯O2^i^	0.84	1.83	2.6597 (19)	170
N1—H71⋯O3^ii^	0.83 (2)	2.10 (2)	2.8963 (18)	161 (2)
C3—H3*B*⋯O3^ii^	0.99	2.51	3.266 (2)	134

Symmetry codes: (i) 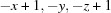; (ii) 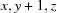.

**Fig. 1.**
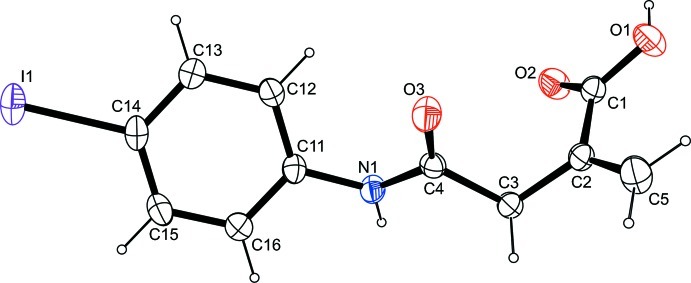
The molecular structure of the title compound, with atom labels and anisotropic displacement ellipsoids drawn at 50% probability level.

**Fig. 2.**
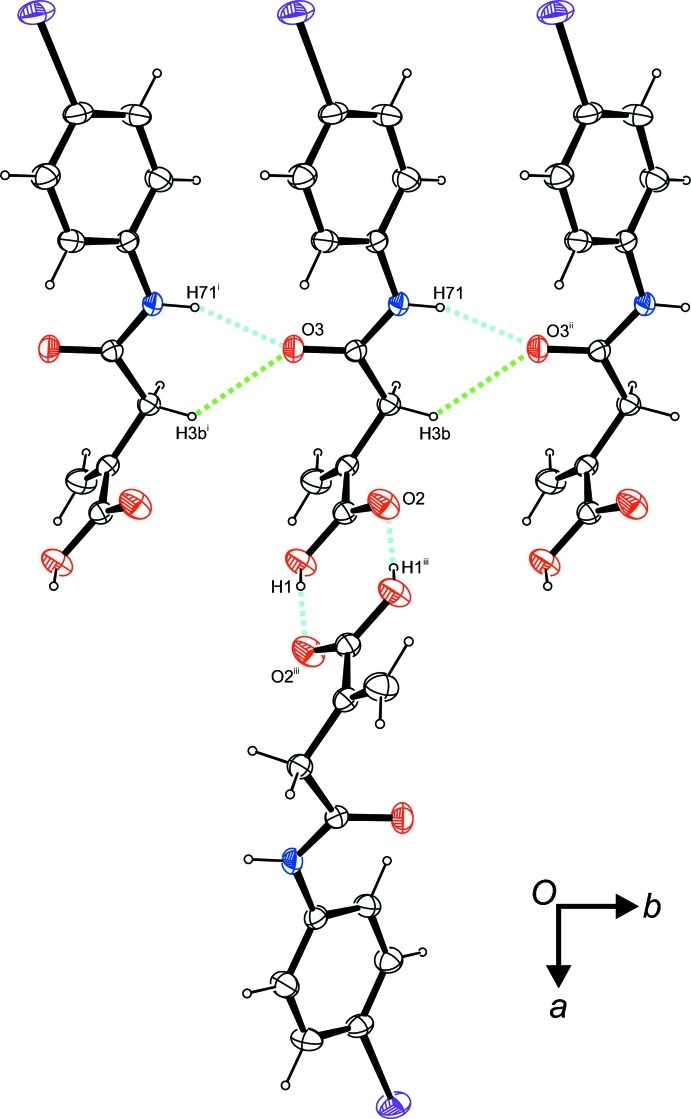
Intermolecular contacts, viewed along [0 0 - 1]. Symmetry operators: ix, y - 1, z; iix, y + 1, z; iii -x + 1, -y, -z + 1.

**Fig. 3.**
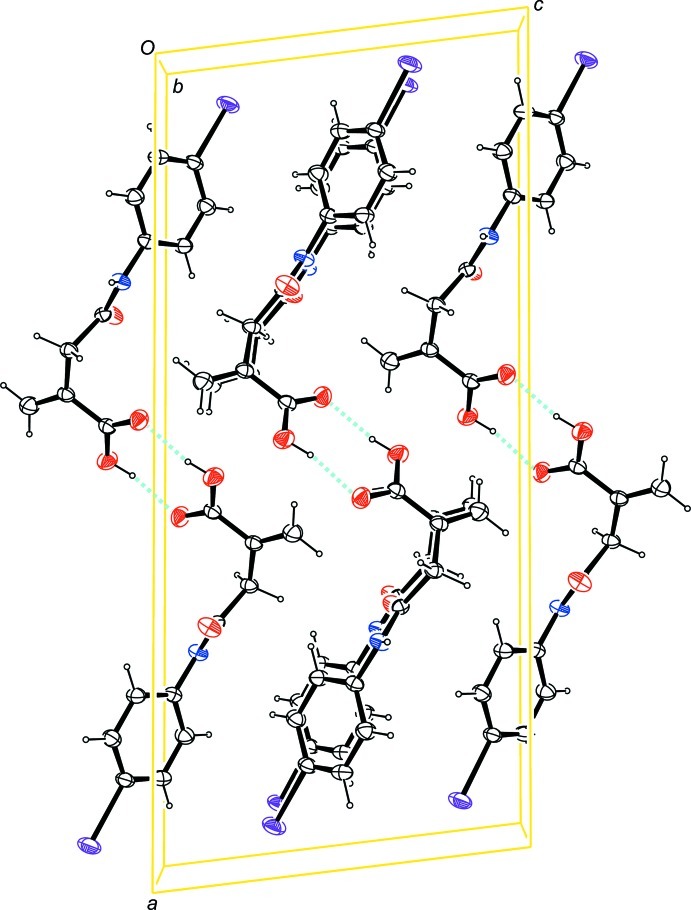
Molecular packing of the title compound, viewed along [0 1 0] (anisotropic displacement ellipsoids drawn at 50% probability level). Blue dashed lines indicate hydrogen bonds between the carboxylic acid groups.

## supplementary crystallographic information

### Comment

Itaconic acid anhydride is a monomeric building block obtained from renewable sources. Copolymers containing both hydrophilic and hydrophobic segments are drawing considerable attention because of their possible use in biological systems. In this aspect, *N*-substituted itaconamic acid derivatives have attracted attention due to their amphiphilic properties. Being more reactive than maleic anhydride, itaconic anhydride has already been applied for introducing polar functional groups into polymers (Oishi, 1980; Urzua *et al.*,1998). In addition, the basic skeleton of itaconic anhydride is useful for the synthesis of cyclic derivatives of imides (Shetgiri & Nayak, 2005), pyridazines (Katla *et al.*, 2011), oxazepines and diazepines (Hanoon, 2011) which show pharmacological activity. In continuation of our studies of pharmacologically active compounds, the crystal structure of the title compound was determined.

The C=C bond is present as its *anti*-Saytzeff tautomer. The N–C(=O) bond length of 1.351 (2) Å is indicative of amide-type resonance. The least-squares plane defined by the atoms of the aromatic moiety on the one hand and the non-hydrogen atoms of the carboxylic acid group on the other hand enclose an angle of 74.82 (11) ° (Fig. 1).

In the crystal, C–H···O contatcs whose range falls by more than 0.1 Å below the sum of van-der-Waals radii are observed next to classical hydrogen bonds of the N–H···O and C–H···O type. The N–H···O hydrogen bonds are supported by the carbonyl oxygen atom of the amide functionality as acceptor. Simultaneously, one of the hydrogen atoms of the methylene group forms a C–H···O contact to the same oxygen atom which, therefore, acts as twofold acceptor. The carboxylic acid groups engage in the common dimeric hydrogen bonding pattern frequently encountered for many carboxylic acids. In total, the molecules are connected to dimeric chains along the crystallographic *b* axis. Metrical parameters as well as information about the symmetry of these contacts are summarized in Table 1. In terms of graph-set analysis (Etter *et al.*, 1990; Bernstein *et al.*, 1995), the descriptor for these contacts is *C*^1^_1_(4)*C*^1^_1_(4)*R*^2^_2_(8) on the unary level. The shortest intercentroid distance between two aromatic systems was found at 4.7863 (11) Å which is about the length of axis *b* (Fig. 2).

The packing of the title compound in the crystal structure is shown in Figure 3.

### Experimental

Itaconic anhydride (0.112 g, 1 mmol) was dissolved in acetone (30 ml) and 4-iodoaniline (0.219 g, 1 mmol) was added in small portions under stirring at room temperature over a timespan of 30 minutes. The mixture turned into a yellow slurry. Stirring was continued for 1.5 h after which the slurry was filtered and the solid obtained was washed with acetone and dried to yield the title compound. Single crystals suitable for the X-ray diffraction study were grown from methanol by slow evaporation at room temperature.

### Refinement

Carbon-bound H atoms were placed in calculated positions (C–H 0.95 Å for aromatic and vinylic carbon atoms, C–H 0.99 Å for methylene groups) and were included in the refinement in the riding model approximation, with *U*(H) set to 1.2*U*_eq_(C). The H atom of the hydroxyl group was allowed to rotate with a fixed angle around the C–O bond to best fit the experimental electron density (HFIX 147 in the *SHELX* program suite (Sheldrick, 2008)), with *U*(H) set to 1.5*U*_eq_(O). The nitrogen-bound H atom was located on a difference Fourier map and refined freely.

### Crystal data

**Table:**

C_11_H_10_INO_3_	*F*(000) = 640
*M**_r_* = 331.10	*D*_x_ = 1.921 Mg m^−^^3^
Monoclinic, *P*2_1_/*c*	Melting point = 447–445 K
Hall symbol: -P 2ybc	Mo *K*α radiation, λ = 0.71073 Å
*a* = 23.1111 (6) Å	Cell parameters from 9343 reflections
*b* = 4.7863 (1) Å	θ = 2.7–28.3°
*c* = 10.4262 (2) Å	µ = 2.79 mm^−^^1^
β = 97.071 (1)°	*T* = 200 K
*V* = 1144.54 (4) Å^3^	Rectangular, colourless
*Z* = 4	0.29 × 0.16 × 0.10 mm

### Data collection

**Table:**

Bruker APEXII CCD diffractometer	2839 independent reflections
Radiation source: fine-focus sealed tube	2612 reflections with *I* > 2σ(*I*)
Graphite monochromator	*R*_int_ = 0.015
φ and ω scans	θ_max_ = 28.3°, θ_min_ = 2.7°
Absorption correction: multi-scan (*SADABS*; Bruker, 2008)	*h* = −30→30
*T*_min_ = 0.503, *T*_max_ = 0.776	*k* = −6→6
12435 measured reflections	*l* = −8→13

### Refinement

**Table:**

Refinement on *F*^2^	Primary atom site location: structure-invariant direct methods
Least-squares matrix: full	Secondary atom site location: difference Fourier map
*R*[*F*^2^ > 2σ(*F*^2^)] = 0.018	Hydrogen site location: inferred from neighbouring sites
*wR*(*F*^2^) = 0.047	H atoms treated by a mixture of independent and constrained refinement
*S* = 1.06	*w* = 1/[σ^2^(*F*_o_^2^) + (0.0202*P*)^2^ + 0.979*P*] where *P* = (*F*_o_^2^ + 2*F*_c_^2^)/3
2839 reflections	(Δ/σ)_max_ = 0.002
150 parameters	Δρ_max_ = 0.73 e Å^−^^3^
0 restraints	Δρ_min_ = −0.61 e Å^−^^3^

### Fractional atomic coordinates and isotropic or equivalent isotropic displacement parameters (Å^2^)

**Table:**

	*x*	*y*	*z*	*U*_iso_*/*U*_eq_	
I1	0.056006 (5)	−0.19408 (3)	0.680940 (13)	0.03784 (5)	
O1	0.48205 (6)	−0.1867 (3)	0.34828 (13)	0.0334 (3)	
H1	0.5045	−0.1852	0.4180	0.050*	
O2	0.43726 (6)	0.1500 (3)	0.44728 (13)	0.0335 (3)	
O3	0.30129 (6)	−0.2166 (2)	0.35760 (14)	0.0301 (3)	
N1	0.26626 (6)	0.2123 (3)	0.39810 (15)	0.0238 (3)	
H71	0.2679 (10)	0.382 (5)	0.380 (2)	0.031 (6)*	
C1	0.43994 (7)	−0.0058 (4)	0.35485 (16)	0.0232 (3)	
C2	0.39444 (7)	0.0026 (3)	0.24115 (16)	0.0225 (3)	
C3	0.34191 (7)	0.1767 (3)	0.25648 (16)	0.0232 (3)	
H3A	0.3198	0.2104	0.1703	0.028*	
H3B	0.3547	0.3601	0.2937	0.028*	
C4	0.30202 (7)	0.0375 (3)	0.34331 (15)	0.0202 (3)	
C5	0.40223 (8)	−0.1299 (4)	0.13366 (18)	0.0330 (4)	
H5A	0.4371	−0.2318	0.1286	0.040*	
H5B	0.3729	−0.1235	0.0614	0.040*	
C11	0.21964 (7)	0.1204 (3)	0.46527 (16)	0.0218 (3)	
C12	0.22773 (7)	−0.0842 (4)	0.55994 (17)	0.0263 (3)	
H12	0.2652	−0.1644	0.5823	0.032*	
C13	0.18104 (8)	−0.1721 (4)	0.62227 (18)	0.0284 (4)	
H13	0.1864	−0.3137	0.6865	0.034*	
C14	0.12659 (7)	−0.0515 (4)	0.58994 (16)	0.0265 (3)	
C15	0.11850 (8)	0.1576 (4)	0.49805 (19)	0.0308 (4)	
H15	0.0813	0.2421	0.4777	0.037*	
C16	0.16521 (8)	0.2432 (4)	0.43571 (18)	0.0290 (4)	
H16	0.1599	0.3867	0.3724	0.035*	

### Atomic displacement parameters (Å^2^)

**Table:**

	*U*^11^	*U*^22^	*U*^33^	*U*^12^	*U*^13^	*U*^23^
I1	0.02465 (7)	0.04793 (9)	0.04370 (9)	−0.00628 (5)	0.01530 (5)	−0.00051 (6)
O1	0.0276 (6)	0.0373 (7)	0.0342 (7)	0.0105 (5)	−0.0003 (5)	−0.0044 (6)
O2	0.0336 (7)	0.0386 (8)	0.0276 (6)	0.0087 (6)	0.0003 (5)	−0.0064 (5)
O3	0.0333 (7)	0.0172 (5)	0.0428 (7)	0.0000 (5)	0.0167 (6)	0.0006 (5)
N1	0.0239 (7)	0.0160 (6)	0.0331 (7)	0.0002 (5)	0.0096 (6)	0.0033 (6)
C1	0.0217 (7)	0.0230 (7)	0.0258 (7)	0.0002 (6)	0.0066 (6)	0.0026 (6)
C2	0.0204 (7)	0.0216 (7)	0.0262 (7)	−0.0017 (6)	0.0060 (6)	0.0036 (6)
C3	0.0205 (7)	0.0223 (8)	0.0274 (8)	0.0007 (6)	0.0050 (6)	0.0052 (6)
C4	0.0179 (7)	0.0194 (7)	0.0230 (7)	−0.0010 (5)	0.0011 (5)	0.0009 (6)
C5	0.0271 (9)	0.0400 (10)	0.0320 (9)	0.0016 (7)	0.0040 (7)	−0.0066 (8)
C11	0.0201 (7)	0.0201 (7)	0.0261 (7)	−0.0020 (6)	0.0064 (6)	−0.0024 (6)
C12	0.0194 (7)	0.0279 (8)	0.0318 (8)	0.0017 (6)	0.0046 (6)	0.0035 (7)
C13	0.0251 (8)	0.0316 (9)	0.0294 (8)	−0.0007 (7)	0.0072 (7)	0.0045 (7)
C14	0.0194 (7)	0.0323 (9)	0.0291 (8)	−0.0046 (6)	0.0083 (6)	−0.0042 (7)
C15	0.0182 (7)	0.0390 (10)	0.0356 (9)	0.0038 (7)	0.0055 (7)	0.0012 (8)
C16	0.0246 (8)	0.0306 (9)	0.0324 (9)	0.0044 (7)	0.0060 (7)	0.0070 (7)

### Geometric parameters (Å, º)

**Table:**

I1—C14	2.0994 (16)	C3—H3B	0.9900
O1—C1	1.311 (2)	C5—H5A	0.9500
O1—H1	0.8400	C5—H5B	0.9500
O2—C1	1.226 (2)	C11—C12	1.387 (2)
O3—C4	1.2258 (19)	C11—C16	1.388 (2)
N1—C4	1.351 (2)	C12—C13	1.392 (2)
N1—C11	1.425 (2)	C12—H12	0.9500
N1—H71	0.83 (2)	C13—C14	1.388 (2)
C1—C2	1.485 (2)	C13—H13	0.9500
C2—C5	1.319 (2)	C14—C15	1.382 (3)
C2—C3	1.497 (2)	C15—C16	1.389 (3)
C3—C4	1.523 (2)	C15—H15	0.9500
C3—H3A	0.9900	C16—H16	0.9500
			
C1—O1—H1	109.5	C2—C5—H5B	120.0
C4—N1—C11	123.75 (14)	H5A—C5—H5B	120.0
C4—N1—H71	117.5 (16)	C12—C11—C16	119.74 (15)
C11—N1—H71	117.8 (16)	C12—C11—N1	121.60 (15)
O2—C1—O1	123.55 (16)	C16—C11—N1	118.65 (15)
O2—C1—C2	120.76 (15)	C11—C12—C13	120.12 (16)
O1—C1—C2	115.69 (15)	C11—C12—H12	119.9
C5—C2—C1	120.52 (16)	C13—C12—H12	119.9
C5—C2—C3	123.74 (16)	C14—C13—C12	119.52 (16)
C1—C2—C3	115.70 (14)	C14—C13—H13	120.2
C2—C3—C4	112.22 (13)	C12—C13—H13	120.2
C2—C3—H3A	109.2	C15—C14—C13	120.72 (16)
C4—C3—H3A	109.2	C15—C14—I1	120.15 (13)
C2—C3—H3B	109.2	C13—C14—I1	119.13 (13)
C4—C3—H3B	109.2	C14—C15—C16	119.43 (16)
H3A—C3—H3B	107.9	C14—C15—H15	120.3
O3—C4—N1	123.00 (15)	C16—C15—H15	120.3
O3—C4—C3	121.66 (15)	C11—C16—C15	120.43 (17)
N1—C4—C3	115.28 (14)	C11—C16—H16	119.8
C2—C5—H5A	120.0	C15—C16—H16	119.8
			
O2—C1—C2—C5	168.65 (18)	C4—N1—C11—C16	−131.82 (18)
O1—C1—C2—C5	−11.0 (2)	C16—C11—C12—C13	2.0 (3)
O2—C1—C2—C3	−9.2 (2)	N1—C11—C12—C13	−179.01 (16)
O1—C1—C2—C3	171.13 (14)	C11—C12—C13—C14	−0.7 (3)
C5—C2—C3—C4	108.2 (2)	C12—C13—C14—C15	−1.0 (3)
C1—C2—C3—C4	−74.03 (18)	C12—C13—C14—I1	178.29 (14)
C11—N1—C4—O3	−7.6 (3)	C13—C14—C15—C16	1.4 (3)
C11—N1—C4—C3	169.68 (15)	I1—C14—C15—C16	−177.91 (14)
C2—C3—C4—O3	−25.0 (2)	C12—C11—C16—C15	−1.6 (3)
C2—C3—C4—N1	157.71 (14)	N1—C11—C16—C15	179.35 (17)
C4—N1—C11—C12	49.1 (2)	C14—C15—C16—C11	−0.1 (3)

### Hydrogen-bond geometry (Å, º)

**Table:**

*D*—H···*A*	*D*—H	H···*A*	*D*···*A*	*D*—H···*A*
O1—H1···O2^i^	0.84	1.83	2.6597 (19)	170
N1—H71···O3^ii^	0.83 (2)	2.10 (2)	2.8963 (18)	161 (2)
C3—H3*B*···O3^ii^	0.99	2.51	3.266 (2)	134

Symmetry codes: (i) −*x*+1, −*y*, −*z*+1; (ii) *x*, *y*+1, *z*.
